# Continuity of Monolayer-Bilayer Junctions for Localization of Lipid Raft Microdomains in Model Membranes

**DOI:** 10.1038/srep26823

**Published:** 2016-05-27

**Authors:** Yong-Sang Ryu, Nathan J. Wittenberg, Jeng-Hun Suh, Sang-Wook Lee, Youngjoo Sohn, Sang-Hyun Oh, Atul N. Parikh, Sin-Doo Lee

**Affiliations:** 1 School of Electrical Engineering #032, Seoul National University, Kwanak P.O. Box 34, Seoul 151-600 Korea; 2 Department of Electrical and Computer Engineering, University of Minnesota, Minneapolis, Minnesota 55455, USA; 3 Department of Anatomy, College of Korean Medicine, Institute of Oriental Medicine, Kyung Hee University, Seoul 130-701, Korea; 4 Department of Biomedical Engineering, University of Minnesota, Minneapolis, Minnesota 55455, USA; 5 Departments of Biomedical Engineering and Chemical Engineering & Materials Science, University of California, Davis, California 95616, USA

## Abstract

We show that the selective localization of cholesterol-rich domains and associated ganglioside receptors prefer to occur in the monolayer across continuous monolayer-bilayer junctions (MBJs) in supported lipid membranes. For the MBJs, glass substrates were patterned with poly(dimethylsiloxane) (PDMS) oligomers by thermally-assisted contact printing, leaving behind 3 nm-thick PDMS patterns. The hydrophobicity of the transferred PDMS patterns was precisely tuned by the stamping temperature. Lipid monolayers were formed on the PDMS patterned surface while lipid bilayers were on the bare glass surface. Due to the continuity of the lipid membranes over the MBJs, essentially free diffusion of lipids was allowed between the monolayer on the PDMS surface and the upper leaflet of the bilayer on the glass substrate. The preferential localization of sphingomyelin, ganglioside GM1 and cholesterol in the monolayer region enabled to develop raft microdomains through coarsening of nanorafts. Our methodology provides a simple and effective scheme of non-disruptive manipulation of the chemical landscape associated with lipid phase separations, which leads to more sophisticated applications in biosensors and as cell culture substrates.

In living cells, membrane-mediated processes depend primarily on the dynamic heterogeneous features of the cellular membrane. Such dynamic membrane heterogeneities are implicated in a variety of cellular functions including membrane trafficking^1^, messenger molecule transport^2^,^3^, domain organization^4^,^5^,^6^, and signaling^7^. They manifest themselves in the creation and remodeling of membrane domains with compositional gradients and activate cellular functions associated with the lateral molecular heterogeneity^1^,^8^,^9^, along with the transverse molecular asymmetry. The membrane heterogeneity may be generated as a result of the local change of the rigidity^10^, the thickness^11^, the packing density^12^, and inter-leaflet interactions^13^ of the membrane through the association of particular lipids and proteins. From the viewpoint of the lipid phase separation, it is very important to explore how the membrane heterogeneity plays a role on the formation of raft domains in a bilayer or a monolayer in the membrane. In this case, the continuity of the membrane from a bilayer to a monolayer, being critical for allowing the free diffusion of the lipids, has been a long-standing challenging issue. However, except for tethered polymer-supported lipid membrane,^14^ such continuous and freely diffusive monolayer-bilayer junction (MBJ) in a supported membrane system has not been fully realized so far.

Several strategies have been implemented to reconstruct the MBJ system *in vitro*. However, in most of the cases, a lipid-free gap was appeared^15^,^16^,^17^,^18^ so that the lipid mixing across the MB boundary was accordingly prohibited. One interesting case is a lipid MBJ system with a self-assembled monolayer produced by microcontact printing^19^ although the lipid mobility across the MBJ and the morphology-dependent chemical patterning in such system were not investigated. In fact, for the assessment of the selective sorting of particular lipids in response to underlying surface properties, a lipid patterning method allowing the lateral molecular mixing across the MBJ with no diffusion barrier is inevitably needed. Two critical geometrical requirements for the continuity of the MBJ are (i) a precise pattern of a biocompatible material and (ii) the height of the pattern (*h*, Fig. 1A) comparable to the thickness of a lipid monolayer (*L*_*t*_, Fig. 1A). In addition, a proper physiochemical condition for the formation of a lipid monolayer should be imposed on the surface energy of the pattern.

Here, we report a simple, versatile, and non-disruptive methodology of producing continuous MBJs based on the patterns of poly(dimethylsiloxane) (PDMS) which is widely used as a biocompatible material to support a single lipid leaflet. Note that a lipid monolayer is formed over a hydrophobic PDMS pattern while a lipid bilayer is over a hydrophilic glass substrate. Depending on the microcontact printing temperature of the PDMS, the surface hydrophobicity (or the surface energy) was varied. The continuity between the lipid monolayer and the bilayer across the PDMS patterns was realized in our MBJ system at a certain printing temperature (200 °C). In the circumstance of the free diffusion of lipids across the MBJs, we observed the preferential localization of sphingomyelin, ganglioside GM1, and cholesterol in the monolayer region and a selective assembly of liquid-ordered (*l*_*o*_) domains, called a raft phase^9^,^20^, within the lipid monolayer through coarsening nanorafts on the PDMS pattern. Based on our findings, as a model for ligand-receptor recognition, we demonstrated the specific binding of cholera toxin subunit B (CTxB) to glycolipid receptor GM1 in the monolayer region.

## Results and Discussions

### Substrate patterning for MBJs

**Figure 1:**
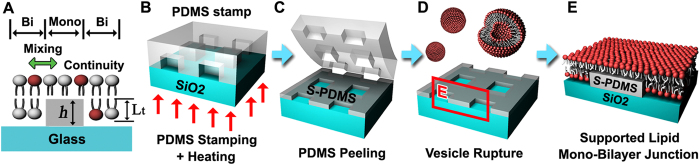
Surface patterning and formation of lipid membranes with MBJs. (**A**) The schematic diagram shows the cross-sectional view of a supported membrane across the MBJ in terms of the thickness of a lipid monolayer (*L*_*t*_) and that of the S-PDMS layer (*h*). (**B)** A glass substrate in contact with the PDMS stamp with desired patterns for transfer printing at 200 °C for 3 min. (**C)** Removal of the PDMS stamp leaving behind grid patterns of the PDMS oligomers onto the glass substrate. (**D,E**) The exposure of the vesicles to the glass with the S-PDMS patterns (**D**) and the formation of a supported lipid membrane through spontaneous vesicular rupture across the MBJs on the S-PDMS patterned glass substrate (**E**). The phospholipids depicted are DOPC (represented by a white head group) and Texas Red-DHPE (red head group).

For the construction of a continuous MBJ between a lipid bilayer and an adjacent monolayer (Fig. 1A), the substrate patterning was carried out in such way that three requirements are met; (i) the generation of both a hydrophilic region and a hydrophobic region to simultaneously form a lipid bilayer and a lipid monolayer, respectively^21^,^22^, (ii) the tuning of the hydrophobicity to ensure the connection of the monolayer with the upper leaflet of the bilayer, and (iii) the thickness match between the pattern and the lower leaflet of the bilayer. In most previous works, the pattern roughness and the topographic mismatch across the hydrophobic and hydrophilic regions positioned underneath a supported lipid membrane were found to render lipid-free gaps^15^,^16^,^17^ at monolayer-bilayer boundaries. In our case, the self-inking PDMS transfer^23^ by thermally-assisted contact printing^24^,^25^ (Fig. 1B) was employed to produce the MBJs with high spatial fidelity on a hydroxylated glass substrate. Our thermally-assisted approach allowed to precisely tune the hydrophobicity (or the surface energy) of the PDMS pattern and the surface topography with the thickness of a few nanometers together with the surface roughness below a nanometer. Residual patterns of the stamped-PDMS (S-PDMS) were remained on the hydroxylated glass^26^,^27^,^28^ after peeling the PDMS stamp from the glass substrate (Fig. 1C). The one-step vesicle fusion process was performed on the hydrophilic glass substrate with hydrophobic S-PDMS patterns to promote the spontaneous formation of the MBJ features (Fig. 1D,E)^29^.

### Characterization of hydrophobic S-PDMS patterns

**Figure 2:**
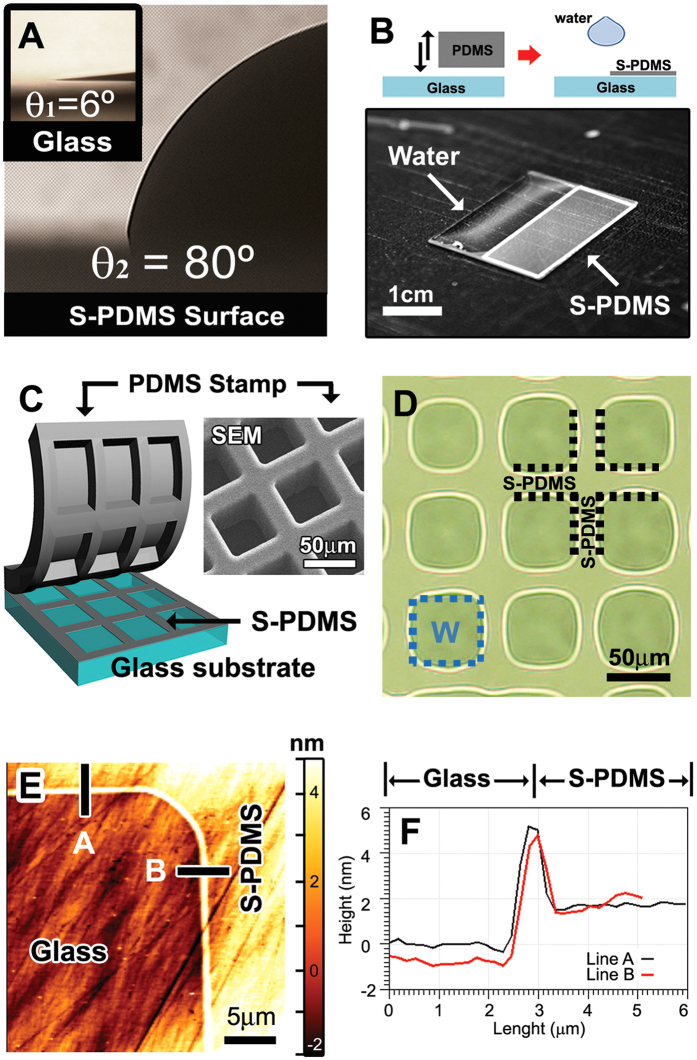
Hydrophobicity of patterned S-PDMS surfaces. (**A**) Microphotographs showing the contact angles of water on a hydroxylated glass surface (*θ*_1_ = 6°, inset) and on the S-PDMS surface transferred at 200 °C for 3 min (*θ*_2_ = 80°). (**B)** A water droplet expelled from the S-PDMS pattern (white rectangle; 1 cm x 2 cm) transferred to a piranha-cleaned glass of 2 cm × 2 cm, showing the distinct wettability boundary along the S-PDMS pattern. The upper inset shows the schematic illustrations of the experiment. (**C**) The stamping process of the PDMS on a glass substrate and the SEM image of the PDMS stamp. (**D**) A microphotograph obtained during the water vapor test on square regions (50 μm × 50 μm each) enclosed by 15 μm-wide PDMS grids shows that water only condenses on the hydrophilic glass regions bounded by S-PDMS. The scale bar is 50 μm. (**E,F)**, The AFM image (**E**) of the glass (non-contact) and S-PDMS (contact) regions, and corresponding thickness profiles (**F**) along two paths, (**A**,**B**) across the boundary between the glass and S-PDMS regions.

Due to reactions of the transferred oligomers with silanols on the glass surface^27^,^28^, the hydrophobic patterns exhibited the long-term stability over 30 days^24^. As shown in Table 1, the contact angle (*θ*_*c*_) of water on the S-PDMS surface increases monotonically with the printing temperature, i.e., the surface energy decreases. Note that the S-PDMS with low-molecular-weight oligomers altered the surface energy from high (hydrophilic) to low (hydrophobic). The transfer of S-PDMS onto the hydroxylated glass surface was reproducible even for multiple processes of transfer printing with the same PDMS stamp as demonstrated in the contact angle stability test^28^. The contact angle of water on a piranha-cleaned glass surface (*θ*_*1*_ = 6°) and that on the S-PDMS surface prepared at 200 °C (*θ*_*2*_ = 80°) were shown in Fig. 2A. As shown in Fig. 2B, water was expelled from the S-PDMS region and confined only within the bare glass region in aqueous environment. Moreover, on the glass substrate with the S-PDMS grid patterns (Fig. 2C), the condensation of water vapor occurred only within the glass surface surrounded by the S-PDMS grids (Fig. 2D), which is consistent with previous results^30^,^31^.

Based on the results obtained using an atomic force microscope (AFM) in the contact mode, the S-PDMS transferred at 200 °C was found to be 3.1 ± 0.8 nm thick (Fig. 2E,F), which was the average over five different positions. This is comparable to a typical thickness of a single lipid monolayer^32^. Sharp ridge boundaries of about 3 nm high were observed at the edges of the transferred lines (Fig. 2F) as in the previous works^30^,^31^,^33^,^34^. Except for the boundaries, the surface roughness of the S-PDMS pattern was 0.7 ± 0.3 nm and that of the glass substrate was 0.6 ± 0.1 nm over five different positions. It should be noted that the roughness difference between the two surfaces is essentially negligible. The transfer of the PDMS oligomers from the PDMS stamp to the glass substrate is accelerated by the thermal energy absorbed during the heat treatment so that the S-PDMS roughness decreases with increasing the stamping temperature^25^,^35^.

### Dependence of the MBJ topography on the surface hydrophobicity

**Figure 3:**
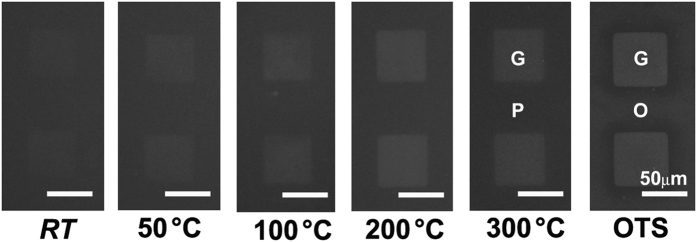
Effect of stamping temperature on lipid membranes formed on S-PDMS patterns. Epifluorescence images of MBJs formed after S-PDMS patterning at increasing stamping temperatures. S-PDMS grids were patterned leaving 50 μm × 50 μm squares of bare glass (**G**) surrounded by S-PDMS (**P**). The MBJ features were formed by rupturing vesicles composed of DOPC with 1 mol % TR-DHPE. As a control experiment, a MBJ was formed on an S-OTS (**O**) grid stamped at room temperature (RT) (rightmost image).

To examine whether our glass substrate with the S-PDMS grid patterns meets the primary requirements for the MBJ, a membrane containing 1,2-dioleoyl-sn-glycero-3-phophocholine (DOPC) doped with 1 mol% of fluorescent Texas Red 1,2-dihexadecanoyl-sn-glycero-3-phosphoethanolamine (TR-DHPE) was formed through spontaneous vesicular rupture. Figure 3 shows epifluorescence images of the MBJs on the glass surface with the S-PDMS patterns produced at various transfer temperatures. As discussed above, the surface energy (γ) of the S-PDMS surface decreases monotonically from 62.0 to 29.2 mJ/m^2^ as the transfer temperature increases from room temperature to 300 °C (see Table 1). It was found that the surface energy of γ = 35.4 mJ/m^2^ for the S-PDMS region, produced at 200 °C for 3 min, was the best case for reconstructing the lipid monolayer formation^36^, ruling out the possibility of forming a bilayer on the S-PDMS.

**Figure 4:**
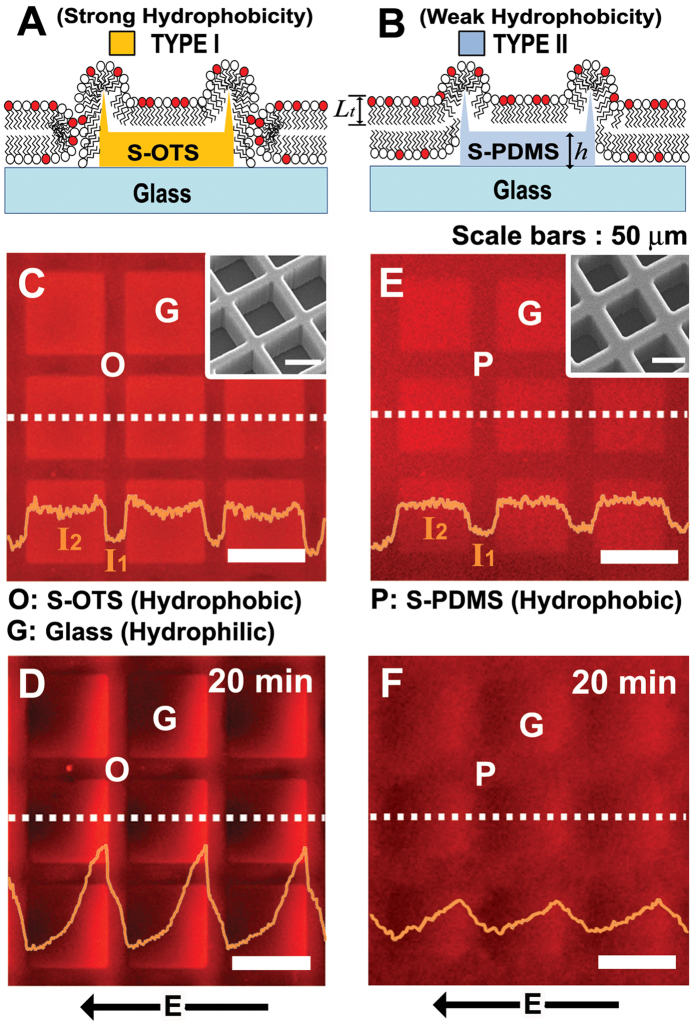
Topography of MBJ on S-OTS and the S-PDMS patterns. (**A**,**B**) Schematic illustrations showing an MBJ with lipid-free gaps at the boundary of S-OTS patterns (TYPE I, **A**) and an MBJ on S-PDMS maintaining the continuity of the upper monolayer (TYPE II, **B**). (**C,D**) Epifluorescence images and the corresponding intensity profiles across the S-OTS (Type I; **C**) and the S-PDMS (Type ΙΙ; **D**) along the white dotted lines (yellow curves; *I*_1_ for P and O regions and *I*_2_ for G region) for a supported phospholipid membrane composed of DOPC and 1 mol% TR-DHPE. The G regions (50 μm × 50 μm) were surrounded by 10 μm-wide S-OTS grids (**C**) or 15 μm-wide S-PDMS grids (**E**). The insets show the SEM images of two PDMS stamps. Epifluorescence images for the supported membrane across the S-OTS (**D**) and the S-PDMS (**F**) after the application of an electric field of 40 V/cm in direct current for 20 min. The yellow lines in (**D**,**F**) are the fluorescence intensity profiles along the while dotted lines. All scale bars are 50 μm.

The formation of continuous MBJs depends critically on the interplay between the membrane tension and surface energy (see details in Supplementary Information Fig. S1). The surface energy of the hydrophobic regions required for the continuous MBJ lies typically in the range of γ  = 19–50 mJ/m^2 ^36. In contrast to the stamped *n*-octadecyltrichlorosilane (S-OTS) surface with ‘strong’ hydrophobicity of γ = 17.6 mJ/m^2^ (Fig. 4A, Type I), the S-PDMS surface must exhibit ‘weak’ hydrophobicity so as to avoid the creation of a hemi-micellar closed lipid edge which generate necessarily the free-gap (or the discontinuity) of the membrane over the MBJ. (Fig. 4B, Type II). From the AFM data, the ridge boundaries of a few nanometers were clearly seen at the edges of the S-PDMS pattern.

To examine the continuity of the MBJs over the hydrophobic patterns (S-OTS vs S-PDMS), an electric field-directed migration test^37^ was performed. As a strong hydrophobic case, grid patterns (each 10 μm wide) of S-OTS (denoted by **O**) were prepared on a hydroxylated glass surface in square (50 μm × 50 μm; denoted by **G**) as shown in Fig. 4C. The thickness of the S-OTS pattern was about 2.2 nm which is comparable to that of a lipid monolayer (*L*_t_). According to the contact angle of water on the S-OTS surface (*θ*_*c *_ ≈ 110°), the hydrophobicity is strong enough to produce a lipid monolayer^38^. Note that the fusion of small unilamellar vesicles (SUVs) over the S-OTS patterned glass is known to produce non-contiguous lipid-free gaps at the MBJ^15^. The binary fluorescence patterns reflect the membrane morphology; the brighter emission for a bilayer membrane and the weaker emission for a monolayer membrane as shown in Fig. 4A. By the application of an electric-field, the negatively charged TR-DHPE lipids migrate electrophoretically toward the anode and are accumulated at the **G**/**O** boundary, suggesting that the monolayer over the S-OTS pattern and the outer-leaflet (monolayer) of the bilayer membrane are physically disconnected (Fig. 4C,D). Upon the application of the electric field of 40 V/cm for 20 min, from the measured intensity profiles of the fluorescence, it is clear that discontinuous behavior of the MBJ across the S-OTS grids was more pronounced. Compared to the fluorescence intensity in **O** regions (*I*_1_), the increase of the fluorescence intensity (*I*_2_) in G regions and its sharp drop at the MBJs indicates that movement of the TR-DHPE lipids were essentially prohibited, which implies the existence of a lipid-free gap.

In contrast, for the case of the S-PDMS (**P**) patterns with less hydrophobicity (*θ*_*c*_ = 80°) (each grid of 15 μm wide and *h* ≈ 3 nm high) on the glass surface, the fluorescence intensity (*I*_2_) was initially uniform in **G** as shown in Fig. 4E and gradually increased through **P** under the electric field of 40 V/cm as shown in Fig. 4F. The fluorescence gradient on the S-PDMS was weak and faint in relative to that on the S-OTS. This is primarily attributed to the accumulation of the fluorescence lipids in the inner leaflet of the bilayer toward the wall of the S-PDMS pattern. Other physical origins may be from two sources; (i) the ridge boundaries of a few nanometers high behave as weak barriers for lateral diffusion of lipids in electrophoretic migration and (ii) the disparity of the diffusive characteristics between the monolayer over the S-PDMS pattern and the outer-leaflet (monolayer) of the bilayer membrane (*D*_bi_ > *D*_mono_ ; Fig. S4, Supplementary Information) may influence the fluorescence gradient distribution. Note that as for the lipid membrane morphology, the patterns of the surface hydrophobicity were reflected in the distribution of the fluorescence intensity. For example, the fact that the measured contrast ratio of the fluorescence intensity, *I*_2_/*I*_1_, was about 1.74 for **G/O** and 1.51 for **G/P** is indeed consistent with the previous results in a bilayer-monolayer composite structure^16^. It should be then emphasized that the presence of the ridge boundaries of a few nanometers high, causing the membrane bending on a nanoscale, will not significantly disrupt the main features of the continuity of the MBJ over the S-PDMS pattern on the glass surface. Instead, the strength of the hydrophobicity required for the formation of a lipid monolayer on the supported pattern plays a critical role in generating the continuous MBJ. As another example showing the contiguous nature of the MBJ over the S-PDMS pattern, we fabricated a combinatorial array composed of three different patterns of **P**, **G**, and **O** that were produced through two successive stamping processes (Fig. S2a, Supplementary Information). The data of the fluorescence intensity across the **G**/**P** boundary were found to agree well with the previous studies^30^,^31^. It is concluded that depending on the strength of the hydrophobicity, a scenario for the formation of a supported lipid monolayer and the continuous MBJ over the S-PDMS pattern is valid as long as the edge effect resulting from the ridge boundaries is not strong enough to break the membrane layer.

### Preferential localization of cholesterol and sphingomyelin in monolayers

**Figure 5:**
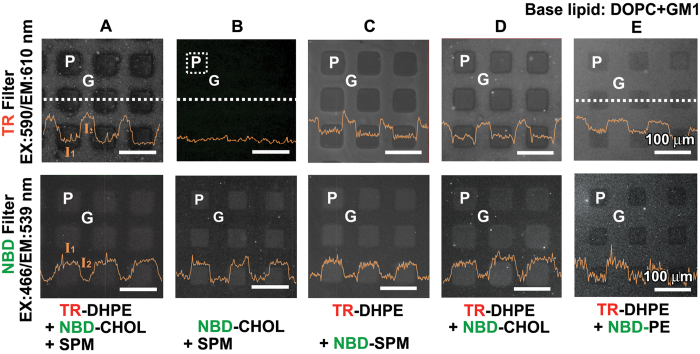
Preferential localization of membrane components in lipid monolayers on S-PDMS patterns. (**A**,**B**) Vesicles with various compositions were exposed to the glass support with the square patterns (50 μm × 50 μm) of S-PDMS. The MBJ features of the membranes composed of DOPC, NBD-CHOL, SPM, and GM1, where TR-DHPE was included (**A**) or excluded (**B**). (**B**–**D**), The MBJ features of the membranes composed of DOPC, TR-DHPE, and GM1, where NBD-SPM (**C**) or NBD-CHOL (**D**) was included. (**E**) The MBJ features of a membrane consisting of DOPC, TR-DHPE, NBD-PE, and GM1. The fluorescence intensities across the membranes along the dotted white lines (yellow curves; *I*_1_ for P region and *I*_2_ for G region) are shown in each micrograph. All scale bars are 100 μm.

We now examine how the S-PDMS patterns and the continuous MBJs modulate the local distributions of different types of lipids (see Table 2 for the membrane compositions). A supported membrane was first prepared on a glass substrate with the S-PDMS patterns in square (each 50 μm × 50 μm) from a mixture (**M1**) containing DOPC and two key raft-partitioning components, sphingomyelin (SPM) and cholesterol (CHOL), together with NBD-labeled cholesterol (NBD-CHOL) and TR-DHPE to facilitate imaging (Fig. 5A). Note that the membrane with such composition is known to phase separate into two co-existing fluidic phases at room temperature^39^, a more dense liquid-ordered (*l*_*o*_) phase enriched with SPM and CHOL and a less dense liquid-disordered (*l*_*d*_) phase consisting primarily of DOPC. In fact, TR-DHPE tends to preferentially partition into the *l*_*d*_ phase while CHOL prefers the *l*_*o*_ phase^40^,^41^,^42^,^43^. This is evident from the fact that the fluorescence of TR-DHPE shows high-contrast patterns between two regions, *i.e.*, brighter for the bilayer region (**G**) and weaker for the monolayer region (**P**). Interestingly, the contrast of the fluorescence in the green channel (EX/EM = 466 nm/539 nm) used for the observation of NBD-CHOL is the opposite. In other words, rather brighter emission was observed in the monolayer region (**P,** *I*_1_) and dimmer emission in the bilayer region (**G,** *I*_2_). To find out if any fluorescence resonance energy transfer (FRET) between two fluorescent tags (TR-DHPE and NBD) occurs^44^, a mixture of lipids without TR-DHPE (**M2**) was also tested (Fig. 5B). The brighter fluorescence emission from NBD-CHOL in the monolayer region suggests that no considerable FRET was involved. The fluorescence profiles, obtained using the two probes in **M1** and NBD-CHOL probe in **M2,** tell us that the raft domain (*l*_*o*_ phase) favors the monolayer region of the MBJ. In additional experiments using NBD-CHOL where the position of the fluorescent moiety was flipped, essentially similar features were obtained. This indicates that the position of the labelled-NBD moiety has no effect on the lateral distribution of CHOL in the MBJ (Fig. S3, Supplementary Information). Two membrane mixtures that contain only one of the two raft components labelled with fluorescent dyes (NBD-SPM or NBD-CHOL), along with CHOL-free **M3** (Fig. 5C) and SPM-free **M4** (Fig. 5D) mixtures, were independently employed on the S-PDMS patterned substrates. The high contrast ratio (*I*_2_/*I*_1_) of about 2.10 for NBD-SPM (Fig. 5C) and that of about 1.85 for NBD-CHOL (Fig. 5D) in **P** (monolayer) imply that both NBD-SPM and NBD-CHOL tend to aggregate in the monolayer rather than in the bilayer. To check the possibility that the preferential localization of SPM and CHOL molecules in the monolayer might originate from NBD molecules, a mixture (**M5)** having NBD-PE was tested. In contrast to the fluorescence distribution patterns by NBD-SPM and the NBD-CHOL, the observed fluorescence intensity was higher in the bilayer than in the monolayer (Fig. 5E), which is similar to the case of TR-DHPE. This leads us to conclude that the raft-partitioning SPM and CHOL molecules themselves prefer to reside in the monolayer over the hydrophobic S-PDMS patterns (**P**) as reported previously^36^.

### Lipid raft growth and receptor-ligand binding

Free lateral diffusion of saturated lipids and cholesterols allows to form the localized packing and the *l*_*o*_ domains in the background of the *l*_*d*_ phase consisting primarily of unsaturated lipids. Phase separation is thought to be modulated as a result of the local change of the rigidity, line tension, thickness, and the packing density of membrane^11^,^12^,^40^,^41^,^42^,^45^. However, much less is known about how a hydrophobic interface at the membrane mid-plane influences the transverse coupling between two leaflets, especially, for the raft formation in the apposing leaflet. We now address how the patterned hydrophobicity comes into play in the selective growth of raft domains via the migration of lipids and the coarsening of raft units across the continuous MBJs. In raft-forming mixture **M6** containing ganglioside GM1, time-lapse microscopy measurements were carried out to monitor the time evolution of *l*_*o*_ domains in lipid monolayers over the S-PDMS and the S-OTS patterns on the glass substrates. The continuity and the fluidity across the MBJ were confirmed by the fluorescence recovery after photobleaching (FRAP) measurements^46^. The diffusion coefficient of TR-DHPE in **M6** mixture in the monolayer over S-PDMS was *D*_mono_ = 0.38 ± 0.1 μm^2^/s, averaged over ten different positions, and that in the bilayer over glass was *D*_bi_ = 1.1 ± 0.1 μm^2^/s (Fig. S4, Supplementary Information).

**Figure 6:**
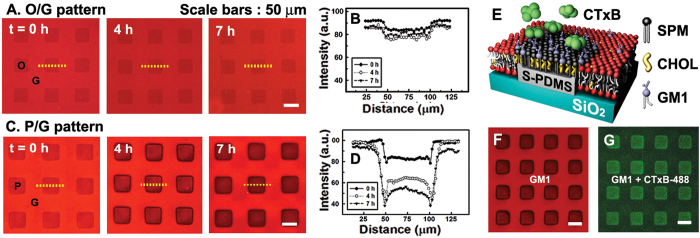
Selective growth of raft domains in lipid monolayers and GM1/CTxB binding. (**A–D)** Epifluorescence images and the corresponding intensity profiles across the S-OTS/glass (O/G) region (**A,B**) and the S-PDMS/glass (P/G) region (**C,D**) along the yellow dotted lines as function of time for a raft-forming lipid mixture (SPM/CHOL/DOPC /GM1/TR-DHPE = 33/33/32/1/1). (**E–G),** Schematic illustration of selective GM1 hetergeneity and the CTxB-GM1 binding process in the lipid raft microarray (**E**). Fluorescence images through Texas Red Filter (**F**) and Alexa Fluor 488 filters (**G**) after incubation of a solution of CTxB-488 for 1 h. All scale bars are 50 μm.

The time course of the *l*_*o*_ domain formation over S-PDMS was directly compared to that over S-OTS as shown in Fig. 6A–D. During several hours, the fluorescence intensity change across the monolayer on S-OTS (**O**; Fig. 6A) was barely noticeable. However, it became quite pronounced in the monolayer on S-PDMS (**P**; Fig. 6B). After 7 h, the intensity of TR-DHPE was greatly diminished in the monolayer on S-PDMS (Fig. 6D). This means that TR-DHPE probes remained mostly in the bilayer and they were strongly excluded from the *l*_*o*_ phase in the S-PDMS regions. In other words, the raft components were diffused across the continuous MBJ and thus resulted in the coarsening of the raft domains^43^ in the monolayer over the S-PDMS.

It should be noted that the *l*_*o*_ phase develops faster near the boundaries than in the center of **P** regions (Fig. 6B). This is because the accumulation of nanorafts, being diffused into the monolayer from the bilayer membrane region, is slowed down due to the mobility difference (*D*_mono_ < *D*_bi_). In addition, the nanoscale curvature at the ridge boundaries facilitates to localize the nanorafts. As the cases in our previous works^41^,^42^, the negative curvatures at the boundaries tend to accelerate the coarsening of the nanorafts. Recently, Collins and Keller^21^ have shown that in giant unilamelar vesicles, the compositional asymmetry across two leaflets can induce or suppress the domain formation, confirming the role of inter-leaflet interactions in the lateral phase-separation of membrane molecules in the absence of proteins. In our MBJ system, the domain formation occurs selectively in the lipid monolayer over the hydrophobic S-PDMS. This is an inter-leaflet effect on the spatial organization of the membrane molecules.

The specific binding of cholera toxin subunit B (CTxB) to glycolipid receptor GM1 was implemented as a model for ligand-receptor recognition (Fig. 6E). The GM1 is known to partition into the *l*_*o*_ phases in model membranes and lipid rafts in cells. The phase-separated lipid membrane with compositional mixture **M6** was incubated with a solution of Alexa Fluor 488 labelled CTxB (CTxB-488; 10 μg/ml) for 1 h (Fig. 6F,G). The strong green fluorescence in **P** regions means that the CTxB-GM1 binding process is highly concentrated in the monolayers. The possibility of the non-specifical binding of the CTxB directly to the S-PDMS regions was ruled out by an additional experiment on the binding of CTxB to GM1-free lipid mixture (Fig. S7, Supplementary Information). The preferential spatial localization of raft-partitioning receptors and the subsequent binding of proteins might have biologically significant implications. For instance, our finding of the GM1 receptors in the monolayer of the *l*_*o*_ phase suggests that the purported role of raft domains as scaffolds in promoting toxin trafficking is more complex than previously thought and might involve the participation of cytoplasmic protein complexes that produce diverse routes for toxin endocytosis^47^,^48^.

## Conclusions

We established a simple, versatile, and non-disruptive method of creating patterns of the lipid monolayers connected continuously with neighboring lipid bilayers. The strategic approach is based on the thermally-assisted transfer printing of the hydrophobic PDMS oligomers onto the hydrophilic glass substrates. The resultant hydrophobicity of the residual S-PDMS region is tunable with the temperature during the transfer printing by a stamp of PDMS. The thickness of the PDMS pattern was about 2–3 nm, which is comparable to that of a monolayer of a typical lipid membrane, and the hydrophobicity was tailored for creating the continuous junctions between lipid monolayers and bilayers. Our work demonstrated an example of a continuous MBJ platform which would be applicable for investigating surface-mediated cell signaling as well as for designing a new class of high throughput biomimetic devices with raft-partitioning or raft-activating membrane components. In previous studies, the sorting of the *l*_*o*_ phase into specific areas is mainly employed by the local change of the membrane properties (such as the curvature^10^, the thickness^11^, and the packing density^12^). However, the roles of the hydrophobicity on the transverse molecular reorganization and membrane perturbations in diverse SLB systems remain poorly understood. This is partly because no reliable and systematic tool for engineering a hydrophobic-patterned surface with high fidelity, allowing the lateral molecular mixing across the MBJs, has been developed so far. Our generic platform may shed light on some intriguing questions such as the physical properties of the lipid mixtures that are not expected to phase separate in GUVs at room temperature but do phase separate in supported membranes. Since it allows the accumulation or depletion of particular lipids over the S-PDMS patterned regions, further studies on the enrichment of the components remain to be carried out as a function of the shape of the S-PDMS pattern, the relative dimension of the S-PDMS pattern to the glass region, temperature, and lipid composition. Finally, the continuous MBJs could be integrated with label-free surface-based detection methods such as surface plasmon resonance and surface-enhanced spectroscopies to monitor the raft-phase forming dynamics^49^.

## Methods

### Biomolecules

1,2-dioleoyl-sn-glycero-3-phophocholine (DOPC) was used in all membranes. Raft-forming mixtures included the sphingomyelin (SPM; brain, porcine) and the cholesterol (CHOL). For fluorescence microscopy, a variety of fluorescent lipid conjugates at trace concentrations (1–3 mol %) were mixed with the primary lipids. The probes used include: (i) Texas red 1,2-dihexadecanoyl-sn-glycero-3-phosphoethanolamine (TR-DHPE, Life Technologies, Carlsbad, CA), (ii) two types of the NBD-labelled cholesterol, 5-cholesten-3ß-ol 6-[(7-nitro-2-1,3-benzoxadiazol-4-yl)amino] caproate (head-labelled NBD-CHOL) and 25-[N-[(7-nitro-2-1,3-benzoxadiazol-4-yl)methyl]amino]-27-norcholesterol (tail-labelled NBD-CHOL), (iii) N-[12-[(7-nitro-2-1,3-benzoxadiazol-4-yl)amino]dodecanoyl]-sphingosine-1-phosphocholine (NBD-SPM; tail-labelled NBD-SPM), and (iv) 1-palmitoyl-2-{12-[(7-nitro-2-1,3-benzoxadiazol-4-yl)amino]dodecanoyl}-*sn*-glycero-3phosphoethano lamine (tail-labeled NBD-PE). All lipids were purchased from Avanti Polar Lipids (Birmingham, Alabama). For protein binding assays, we used monosialoganglioside (GM1; brain, ovine-ammonium salt) and Alexa Fluor 488-labelled cholera toxin B subunit (CTxB-488, Life Technologies, Carlsbad, California).

### Fabrication of PDMS stamps

Before coating the photoresist, a glass substrate was immersed in acetone for 30 min followed by the immersion in deionized (DI) water for 10 min. Both cleaning steps were carried out in an ultra-sonicator at room temperature. After drying the substrate with compressed N_2_ gas, negative photoresist (SU-8 2050, MICROCHEM) was spin-coated onto the glass substrate according to the vendor’s instructions. The substrate was aligned and exposed through a photo-mask using a standard photolithography mask aligner (MA-6, EVG). After the exposure process, photoresist was developed and chemically etched to produce photoresist patterns of 50 μm high. An elastomeric material, Sylgard 184 silicone elastomer (Dow Corning Corp.), mixed with a curing agent in 10:1 ratio by weight, was poured into the photoresist mold and cured for >3 h at 80 °C.

### Deposition of S-PDMS patterns

Glass substrates were rendered hydrophilic by immersing each substrate in the piranha solution (3:1 (v/v) H_2_SO_4_:H_2_O_2_) at 120 °C for 10 min followed by ultra-sonication in DI water for 10 min additionally. The PDMS stamp prepared as above was placed on the cleaned glass substrate. The sandwiched substrate was heated on a hot plate at 200 °C for 3 min and the PDMS stamp was then removed. This thermally-assisted stamping process (at the elevated temperature of 200 °C) allowed the transfer of low-molecular weight PDMS oligomers from the PDMS stamp to the glass substrate^24^,^26^,^27^,^28^. A scanning electron microscope (SEM; XL30FEG, Philips) was used to observe the pattern structures of the PDMS stamp and an atomic force microscope (AFM; AutoProbe CP, Park Scientific) was employed for the surface topographic characterization.

### Preparation of supported membranes

Solutions of the lipids and their desired mixtures (Table 1) in chloroform were first prepared. Using the rapid solvent exchange method^50^, chloroform was evaporated and the lipids were hydrated by Tris buffer (100 mM NaCl and 10 mM Tris at pH. 7.4) simultaneously in a single step to produce an aqueous stock solution. Small unilamellar vesicles (SUVs) from the stock were then produced by extruding 21 times through a 50 nm-filter and incubated on freshly prepared substrate surfaces for 5 min. Subsequently, the excess buffer was replaced by DI water in multiple washing steps to ensure that residual, unfused SUVs were fully removed. The CTxB-488 was used as a raft-maker at the concentration of 10 μg/ml in Tris buffer (100 mM NaCl and 10 mM Tris at pH = 7.4). Fluorescence imaging was carried out using an inverted epifluorescence microscope (Eclipse E600-POL, Nikon) excited at appropriate wavelengths (excitation at 590 nm and emission at 610 nm for Texas Red, excitation at 500 nm and emission at 526 nm for Alexa Fluor 488, and excitation at 466 nm and emission at 539 nm for NBD, 10 × objective) and a He-Ne laser. All the experiments were carried out at room temperature.

### Deposition of S-OTS patterns

For the preparation of *n*-octadecyltrichlorosilane (OTS) patterns on the PDMS surface, the PDMS stamp was dipped into a 10 mM solution of OTS in toluene for 5 min at room temperature, followed by the immersion in toluene (100%) for 10 min additionally to wash away the excess OTS. After blowing the PDMS stamp with N_2_ gas, the OTS-coated PDMS was dried on a hot plate for 5 min to remove any residual toluene from the PDMS stamp. All the S-OTS-inked stamps were in contact with a hydrophilic glass substrate for 3 min at room temperature.

### Electric field experiments and image analysis

Platinum wires were used as electrodes. The electric field of 40 V/cm in direct current generated from a standard power supply was used for the lipid electrophoresis experiments. The details of the experimental setup are described in ref. 37. The diameter and the length of platinum wires were 7 mm and 5 cm, respectively. The gap between two electrodes was 1.5 cm. Data analysis was performed using the software of Image*J*, NIH, USA.

### Determination of surface tension at solid-liquid interface

Consider Young’s equation (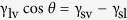) describing the thermodynamic equilibrium of the surface tensions for a droplet of liquid on a solid surface and an equation of state for the interfacial tensions, both of which can be expressed in terms of three surface tensions (γ_sv_, γ_sl_, and γ_lv_ where the subscripts sv, sl, and lv refer to the solid-vapor, the solid-liquid, and the liquid-vapor interfaces, respectively) and the contact angle *θ*. Combining Young’s equation with the equation of state given by eq. 1 27.


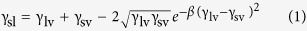


with an empirical parameter *β*, we have the following equation 2:


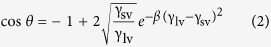


If *θ* is determined from the contact angle measurement, γ_sv_ can be readily estimated from the literature values of *β* = 0.0001247 (mJ/m^2^)^−2^ and γ_lv_ = 72.8 mJ/m^2^ for water. Accordingly, γ_sl_ can be simply determined from Young’s equation with the help of the values of γ_lv,_ γ_sv_, and *θ*.

## Additional Information

**How to cite this article**: Ryu, Y.-S. *et al*. Continuity of Monolayer-Bilayer Junctions for Localization of Lipid Raft Microdomains in Model Membranes. *Sci. Rep.* **6**, 26823; doi: 10.1038/srep26823 (2016).

## Supplementary Material

Supplementary Information

## Figures and Tables

**Table 1:** The contact angle (*θ*_*CA*_) of water (H_2_O) and the surface energy (γ_*sl*_) on different surfaces.

**Surface**	**H**_**2**_**O** ***θ***_***CA***_ **(deg)**	γ_***sl***_ **(mJ/m**^**2**^)
Piranha cleaned glass	< 6.0	72.4
S-PDMS glass - *RT*	36.0	62.0
S-PDMS glass – 50 °C	45.0	56.6
S-PDMS glass – 100 °C	48.5	54.6
S-PDMS glass – 150 °C	60.6	47.5
S-PDMS glass – 200 °C	80.1	35.4
S-PDMS glass – 250 °C	84.0	33.0
S-PDMS glass – 300 °C	90.1	29.2
Bulk PDMS	105.6	19.7
OTS	109.1	17.6

The contact angle of water in each row is the average value over 5 different measurements. The corresponding surface tension (γ_*sl*_) was calculated from Young’s equation combined with an equation of state for interfacial tensions^27^. (see Experimental Methods).

**Table 2:** The compositions of different membranes.

**SLB**	**Membrane composition (mol%)**
**M1**	SPM/NBD-CHOL/CHOL/DOPC/GM1/TR-DHPE = 33/3/30/32/1
**M2**	SPM/NBD-CHOL/CHOL/DOPC/GM1 = 33/3/30/33/1
**M3**	NBD-SPM/SPM/DOPC/GM1/TR-DHPE = 3/30/65/1/1
**M4**	NBD-CHOL/CHOL/DOPC/GM1/TR-DHPE = 3/30/65/1/
**M5**	NBD-PE/PE/ DOPC/GM1/TR-DHPE = 3/30/65/1/1
**M6**	SPM/CHOL/DOPC/GM1/TR-DHPE = 33/33/32/1/1
