# Supplementary material for: Continuity of Monolayer-Bilayer Junctions for Localization of Lipid Raft Microdomains in Model Membranes
Source: Sci Rep. 2016 May 27;6:26823. doi: 10.1038/srep26823 (PMC4882513; doi:10.1038/srep26823)
Supplement: Supplementary Information [file srep26823-s1.pdf]

Supplementary Information:

Continuity of Monolayer-Bilayer Junctions for Localization of Lipid Raft Microdomains in Model Membranes

Yong-Sang Ryu,[1†] Nathan J. Wittenberg,[2] Jeng-Hun Suh,[1] Sang-Wook Lee,[1‡] Youngjoo Sohn,[3], Sang-Hyun Oh,[2, 4] Atul N. Parikh,[5] and Sin-Doo Lee[1]*

[1]School of Electrical Engineering #032, Seoul National University, Kwanak P.O. Box 34, Seoul 151-600, Korea.

[2]Department of Electrical and Computer Engineering, University of Minnesota, Minneapolis, Minnesota 55455, USA

[3]Department of Anatomy, College of Korean Medicine, Institute of Oriental Medicine, Kyung Hee University, Seoul 130-701, Korea.

[4]Department of Biomedical Engineering, University of Minnesota, Minneapolis, Minnesota 55455, USA

[5]Departments of Biomedical Engineering and Chemical Engineering & Materials Science, University of California, Davis, California 95616, USA.

*Address correspondence to sidlee@plaza.snu.ac.kr

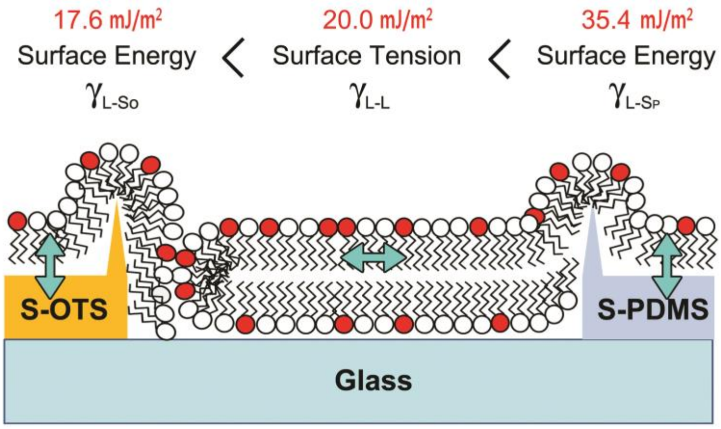

Figure S1. Competition between surface energy and surface tension governs the topologies of the membranes at the MBJs. We calculated the surface energy of the S-PDMS as function of the stamping temperature for fixed time duration (t = 3 min). For the construction of a continuous MBJ, the membrane-substrate surface energy (γ) and height (h) of the S-PDMS pattern should lie in a suitable range (γ = 19-50 mJ/m^2; h = 2–3 nm)[1] to form a lipid monolayer membrane. Transfer of S-PDMS at 200 ℃ for 3 min turned out to be the best case for the continuous MBJ. It is reasonable to consider the physical origin of the continuous MBJ formation as the competition between 'the surface energy' (or the membrane-substrate adhesion force; $\gamma_{L\text{-}S}$) and 'the lipid-to-lipid membrane tension' ($\gamma_{L\text{-}L}$) within the bilayer membrane. As an example, for the case of a relatively strong surface energy on the S-PDMS surface ($\gamma_{L\text{-}Spdms}$), the outer-leaflet of the bilayer will be uniform and continuous since $\gamma_{L\text{-}L} > \gamma_{L\text{-}Spdms}$. For the S-OTS case with a relatively weak surface energy of $\gamma_{L\text{-}Sots}$, however, $\gamma_{L\text{-}L} > \gamma_{L\text{-}Sots}$ so that the lipid membrane breaks down and a lipid-free gap appears naturally. The surface tension between lipid molecules[2] was reported to be approximately 20 mJ/m^2. The membrane-substrate surface energies, 35.4 mJ/m^2 on S-PDMS and 17.6 mJ/m^2 on S-OTS, were determined from the values of the water contact angles (See the Table 1).

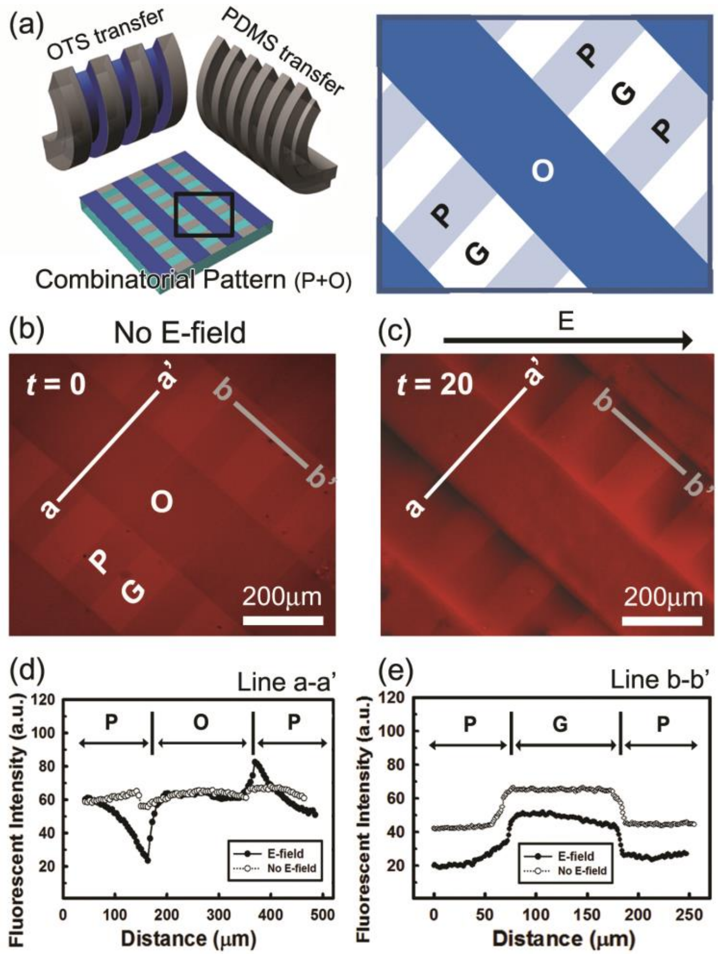

Figure S2. A combinatorial array composed of three types of the regions (**P**, **O**, and **G**) with different strengths of the hydrophobicity produced by successive transfer printing processes in series. **A.** Schematic illustrations of two successive transfer processes of PDMS (**P**) and OTS (**O**) in mutually orthogonal directions on a hydrophilic glass (**G**) substrate, together with the top view of the array (each region of **O, P,** and **G** are 200 μm, 100 μm, 100 μm wide, respectively, and both **P** and **G** are 200 μm long). **B, C,** Epifluorescence images of a supported phospholipid binary membrane (DOPC/TR-DHPE=99/1) formed on the combinatorial array before (**B)** and after (**C)** the application of the electric field *E* = 40 V/cm for 20 min. The black arrow denotes the direction of *E*. **D, E,** Corresponding cross-sectional intensities of the fluorescence measured along two lines of a-a' **(D)** and b-b'**(E)** before (open circles) and after (filled circles) the application of the electric field. Note that the abrupt change of the intensity across the hydrophobic-hydrophobic **(P-O)** boundary represents the discontinuity of the lipid membrane, which is an indicative of the existence of 'a lipid-free gap', prohibiting the lipid mixing. The smooth stepwise intensity change across the hydrophobic (**P)**-hydrophilic (**G**) boundary even under the electric field indicates the contiguous nature of the lipid membrane at the MBJ (**E**). The field diffusion test revealed that charged TR-DHPE lipids were accumulated toward the hydrophobic-hydrophobic **P/O** boundaries (**B-D**). However, no considerable accumulation of the TR-DHPE lipids around the hydrophilic-hydrophobic **G/P** boundaries was observed (**E**).

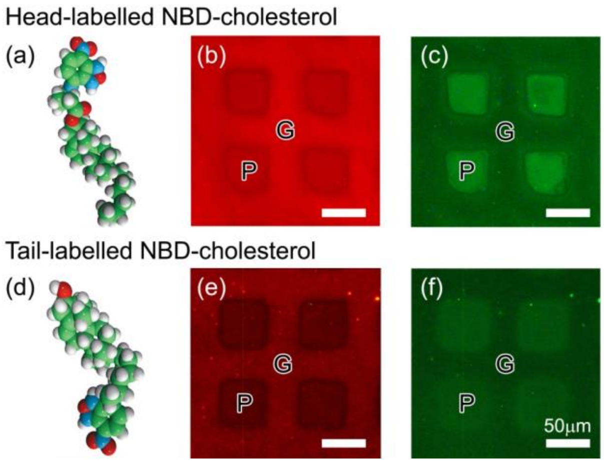

Figure S3. Distribution of the NBD-labelled CHOLs on S-PDMS patterned surfaces. The same composition of the lipid mixture (DOPC/CHOL/NBD-CHOL/TR-DHPE/GM1=65/3/30/1/1) was used in two cases we studied. **A-C,** Molecular structure of the head-labelled NBD-CHOL (**A**). Epifluorescence micrographs of TR-DHPE (**B**) and the head-labelled NBD-CHOL (**C**) in the same supported membrane. **D-F,** Molecular structure of the tail-labelled NBD-CHOL (**D**). Epifluorescence micrographs of TR-DHPE (**E**) and the tail-labelled NBD-CHOL (**F**) in the same supported membrane. The position (head or tail) of the NBD moiety was found to make essentially no difference in the localization of the CHOLs in the MBJ architecture.

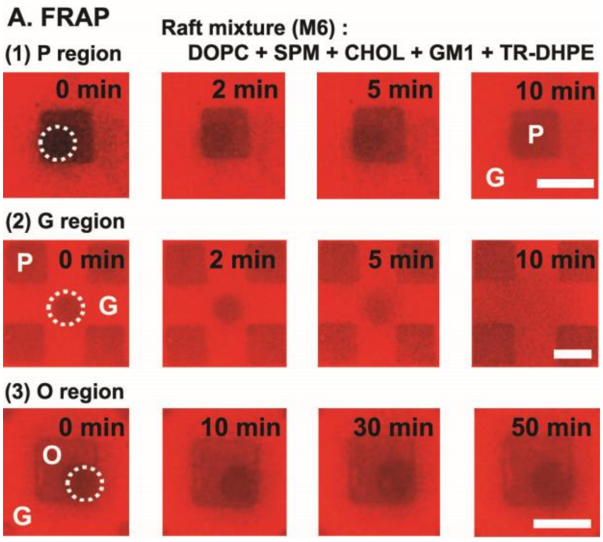

Figure S4. FRAP results for the TR-DHPE molecules on P (A.1), G (A.2) and O (A.3) regions bleached with UV light (a white dotted circle) for 1 min. The continuity and the fluidity across the MBJ were confirmed by the fluorescence recovery after photo-bleaching (FRAP) measurements[3]. For the membrane mixture with raft-components (SPM/CHOL//DOPC/GM1/TR-DHPE =33/33/32/1/1), the diffusion constant of TR-DHPE in the monolayer over the S-PDMS pattern was 0.38 ± 0.1 $\mu m^2/s$ (mean ± s.d., n = 10) and that in the bilayer over the glass substrate was 1.1 ± 0.1 $\mu m^2/s$. All scale bars are 50 μm.

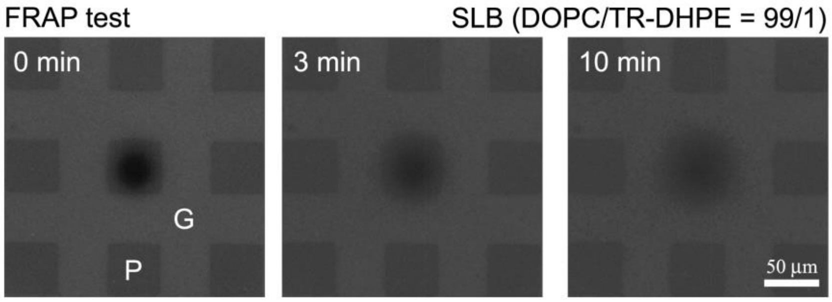

Figure S5. **FRAP experiments showing the radially symmetric nature of the molecular diffusion over the MBJ.** In addition to the electric field-directed migration test shown in Fig. 4, additional FRAP tests in the absence of an electric field were performed to address the issue of the continuity of the lipid membrane across the ridge boundaries at the edges of the S-PDMS patterns. A membrane mixture (DOPC/TR-DHPE=99/1) was deposited over the S-PDMS (P) patterns on the glass (G) substrate by vesicular rupture. The fluorescent lipids in the S-PDMS pattern were selectively photo-bleached in a small (~30 mm) spot (left panel). At the elapsed time of 10 min after photo-bleaching, the lipids in the bleached region were diffused across the MBJs (right panel), confirming the membrane continuity.

.

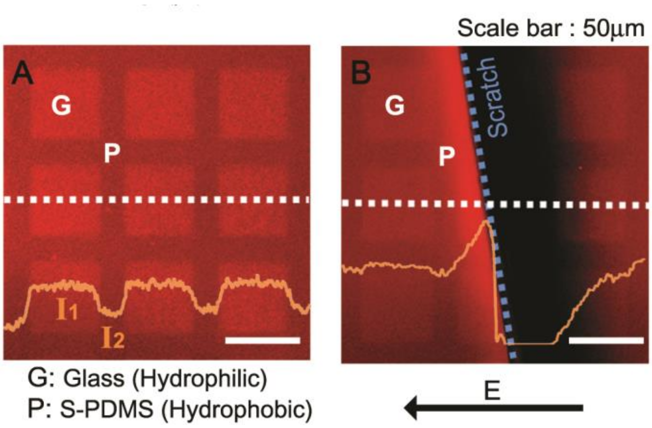

Figure S6. The electrophoretic migration test showing the uniform gradient of the fluorescence of lipids across the MBJ in the S-PDMS pattern. Fluorescent micrographs in the absence (**A**) and presence (**B**) of a lateral electric field (35V/cm) in the direction parallel the substrate. The relative fluorescent intensity profiles (the yellow curves) were measured along the white dotted lines. A mechanical scratch was intentionally employed for producing a geometrical barrier for diffusion in B. The membrane mixture (DOPC/TR-DHPE=99/1) was deposited over the glass (G) substrate with the S-PDMS (P) patterns. Clearly, no considerable defect at the ridge boundaries in the S-PDMS pattern (or across the MBJ) was observed on a macroscopic scale.

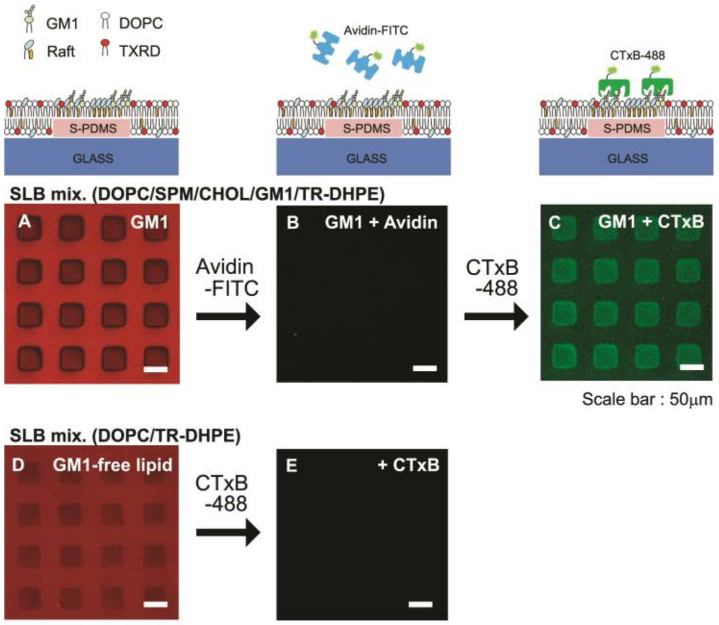

Figure S7. Specific binding of cholera toxin to GM1 in monolayer lipid rafts. A membrane mixture (SPM/CHOL/DOPC/GM1/TR-DHPE = 33/33/32/1/1), known to develop raft domains, was used for the experiments. **A, B,** Epifluorescence micrographs taken after the phase separation of the GM1-containing lipid membrane showing the distribution of TR-DHPE and formation of raft domains in the monolayers over S-PDMS (**A**). Exposure to 50 nM FITC-labelled Avidin for 1 h showed that Avidin did not bind the membrane (**B**). **C**, Epifluorescence micrograph showing the selective binding of CTxB to raft domains in square (10 μg/ml CTxB-488 for 1h, followed by DI water injection). **E**, We also performed a control experiment using GM1-free lipids. No clear indication of the selective binding observed in the fluorescent intensity over the MBJ verifies that CTxB does not bind non-specifically to P regions due to the screening effect of the supported lipid bilayer against non-specific binding between the hydrophobic P region and the hydrophobic ingredient of CTxB.

References

1. Park, J.W. & Lee, G.U. Properties of mixed lipid monolayers assembled on hydrophobic surfaces through vesicle adsorption. *Langmuir* **22**, 5057-5063 (2006).
2. Feller, S.E., Zhang, Y.H. & Pastor, R.W. Computer-simulation of liquid/liquid interfaces .2. surface-tension area dependence of a bilayer and monolayer. *J. Chem. Phys.* **103**, 10267-10276 (1995).
3. Yee, C.K., Amweg, M.L. & Parikh, A.N. Membrane photolithography: Direct micropatterning and manipulation of fluid phospholipid membranes in the aqueous phase using deep-UV light. *Adv. Mater.* **16**, 1184-1189 (2004).
